# Using Multiple Phenotype Assays and Epistasis Testing to Enhance the Reliability of RNAi Screening and Identify Regulators of Muscle Protein Degradation 

**DOI:** 10.3390/genes3040686

**Published:** 2012-11-02

**Authors:** Susann Lehmann, Freya Shephard, Lewis A. Jacobson, Nathaniel J. Szewczyk

**Affiliations:** 1 Medical Resarch Council/Arthritis Research UK Centre for Musculoskeletal Ageing Research, University of Nottingham, Royal Derby Hospital, Derby, DE22 3DT, UK; 2 Medical Resarch Council/Arthritis Research UK Centre for Musculoskeletal Ageing Research, University of Nottingham, School of Veterinary Medicine & Science, Sutton Bonington, LE12 5RD, UK; E-Mail: Freya.Shephard@nottingham.ac.uk; 3 Department of Biological Sciences, University of Pittsburgh, Pittsburgh, PA 15260, USA; E-Mail: ljac@pitt.edu

**Keywords:** RNAi, systems Biology, network Biology, functional Genomics, muscle, proteolysis, *C. elegans*

## Abstract

RNAi is a convenient, widely used tool for screening for genes of interest. We have recently used this technology to screen roughly 750 candidate genes, in *C. elegans*, for potential roles in regulating muscle protein degradation *in vivo*. To maximize confidence and assess reproducibility, we have only used previously validated RNAi constructs and have included time courses and replicates. To maximize mechanistic understanding, we have examined multiple sub-cellular phenotypes in multiple compartments in muscle. We have also tested knockdowns of putative regulators of degradation in the context of mutations or drugs that were previously shown to inhibit protein degradation by diverse mechanisms. Here we discuss how assaying multiple phenotypes, multiplexing RNAi screens with use of mutations and drugs, and use of bioinformatics can provide more data on rates of potential false positives and negatives as well as more mechanistic insight than simple RNAi screening.

## 1. *C. elegans* as A Model for Probing Genomic Control of Muscle Protein Degradation

*Caenorhabditis elegans* is a free-living nematode [1] that is also an established laboratory animal in which genetics and genomics [2,3] can be used to understand cell biology and physiology [4]. For example, this worm has been developed into a model for understanding the intercellular and intramuscular signals that regulate muscle protein degradation [5]. Here we review the approach that we have taken in combining functional genomics techniques with the existing *C. elegans* muscle protein degradation model and discuss some strengths and limitations of combining RNAi with mutations and highly specific pharmacological inhibitors.

## 2. *C. elegans* as RNAi Model

Since the landmark demonstration, in *C. elegans*, that dsRNA can be used to silence gene expression in metazoans [6], dsRNA methods (RNAi, siRNA, shRNA, *etc.*) have gained widespread use in identifying and studying gene function. This has led to *C. elegans* becoming used to help understand how dsRNA, which works very well in *C. elegans*, can be utilized more effectively in other metazoans [7]. In the laboratory, *C. elegans* is typically fed *E. coli* as food source [8]. Therefore, it is common to deliver RNAi by feeding of bacterial vectors expressing large (200 bp to full gene size) dsRNA sequences corresponding to a gene of interest [9]. This feeding of engineered bacteria is less labour intensive than the required *in vitro* synthesis of dsRNA or siRNA prior to gene knockdown in *D. melanogaster* or mammalian cell culture. Two libraries of bacteria that contain dsRNA against roughly each gene in the genome are commercially available. One of these libraries, which uses nearly full-gene cDNA sequences, is fully sequence verified [10] and the other library, which uses genomic sequences ≥200 bp but less than full genes, is roughly 80% sequence verified [11]. So far as we know, there has been no systematic study of the relative efficacies of dsRNA with and without intron sequences. Data for published RNAi experiments utilizing these clones can be accessed via the *C. elegans* community database (www.wormbase.org, [12]) or the RNAi database (www.rnai.org, [13]). Mutations in several genes associated with RNAi efficacy [7,14] have been isolated which allows RNAi to be used in mutants that are more, or less, sensitive to RNAi by feeding. Recent developments also allow feeding vectors to be used to achieve tissue specific gene knockdown [15] and/or knockdown of two genes at once [16]. 

Past use of RNAi by feeding has demonstrated that for developmental and/or behavioural phenotypes (e.g. defects at the level of the whole animal), the false positive rate is very low (<1%) while the false negative rate is potentially substantial (at least 30%) [11]. The low false positive rate in *C. elegans* may be due to the fact that the transcripts produced by the bacteria encode a longer fragment of the gene to be knocked down (200-2000bp) compared to siRNA approaches in other systems (which commonly target only short 21-nucleotide segments of mRNA [17]). These long dsRNA sequences likely give rise to a diverse set of 21–25 nt siRNA sequences *in vivo*, thereby making “off-target effects” (unintentional knockdown of a different but related gene) a much smaller concern than when using small dsRNA [18]. It may also be significant that *C. elegans* lacks an acquired immune system or interferon response, the latter of which is induced by dsRNA in mammalian cells [19] and which can mask the effect of gene knockdown [20]. In contrast, the relatively high false negative rate for RNAi experiments in *C. elegans* may reflect variability in execution of the RNAi protocol, inter-experimenter differences in level of skill or comfort scoring the phenotype in question (see section 5 below), and/or variability in the response to RNAi in different individual worms and/or in worms with different genetic backgrounds. It is therefore important to incorporate quality control measures when performing large scale RNAi screens. Given the potentially high level of irreproducibility with RNAi by feeding, the use of a positive control has been advocated [9]. In addition to the potentially high false negative rate, there are other limitations to using RNAi by feeding in *C. elegans*. For example, it is not currently possible to deliver known or reproducible amounts of dsRNA using the feeding vectors, nor to achieve uniform levels of gene product knockdown *in vivo.*

## 3. RNAi Screening to Identify Genes Potentially Regulating Muscle Protein Degradation

The *C. elegans* model used to study protein degradation [5] utilizes a *lacZ* reporter transgene, with expression driven by an *unc-54* (myosin heavy chain) promoter-enhancer. The fusion protein contains amino acid residues encoded by the first four exons of *unc-54*, and forms active tetramers that do not associate with the sarcomeres, but remain soluble in the muscle cytosol [21]. Expression of this construct switches off upon reaching adulthood, thus stopping synthesis of the reporter protein, but the reporter is stable in well fed wild-type animals for up to a further 72-96 hours [21]. Presence of an active gene product is visualized by histochemical staining where lack of stain reports on lack of LacZ (β-galactosidase) activity, presumably due to protein degradation. Degradation has been confirmed and quantitated by Western blot for starvation [21,22], functional denervation due to neurodegeneration [23,24], and in a number of mutants [25,26,27,28].

**Figure 1:**
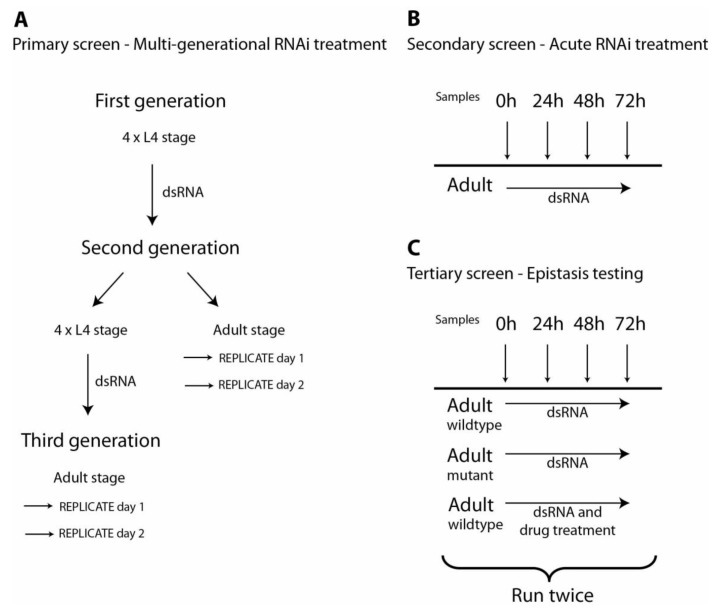
Schematic of the protocol for primary, secondary and tertiary RNAi screens. (A) Flowchart for the chronic, primary RNAi screen over three generations. Four L4 stage animals and their subsequent generation of progeny were fed RNAi throughout their development. Four L4 stage worms of the second generation were transferred to freshly prepared RNAi plates to screen a third generation fed RNAi throughout development. Replicate samples of 20 worms each were examined at day 1 and day 2 of the adult stage for both second and third generations. These animals were scored for developmental and behavioural defects and subcellular defects at each time point. (B) Flowchart for the acute, secondary RNAi screen in adult worms. Genes identified in the chronic screen (panel A) were re-examined in the secondary screen. L1 larvae were synchronized and then synchronized adult worms were fed RNAi. Samples were taken at 0, 24, 48 and 72 hours after onset of feeding and animals were examined for the same subcellular defects as identified in the primary screen. (C) Flowchart for the tertiary RNAi screen. Only genes identified in the acute screen (panel B) as potential regulators of protein degradation were re-examined in the tertiary screen. L1 larvae were synchronized and then synchronized adult worms were fed RNAi. For the tertiary screen, both wild-type and various mutant worms (typically *unc-51*, *daf-18*, and *mpk-1* [30]) were used. Additionally, some wild-type worms were also treated with drugs at the onset of RNAi feeding (typically proteasome inhibitor MG132 [30]). The tertiary screen was run twice for each gene of interest and at least once more if results from the first two runs were not consistent with each other.

**Figure 2:**
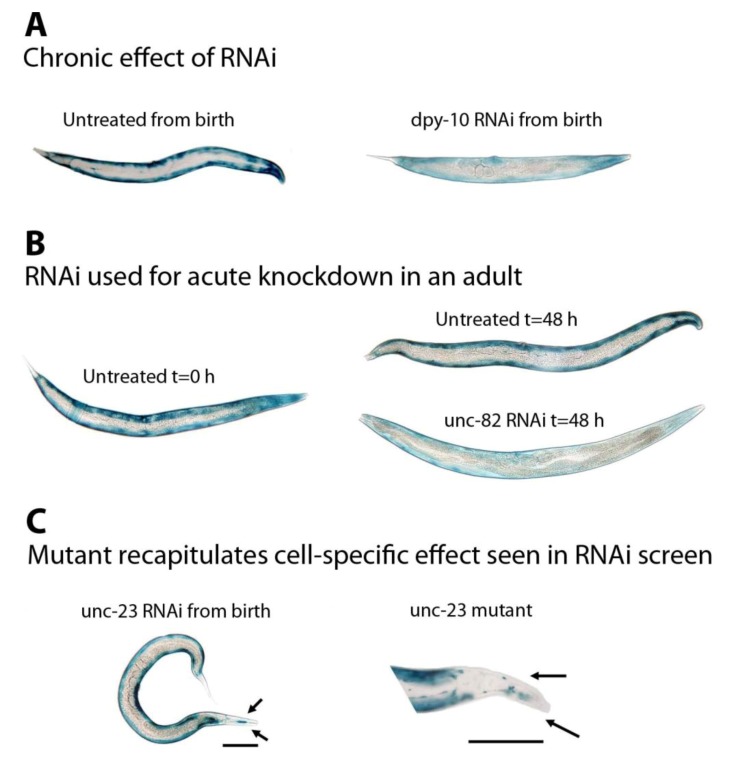
Examples of use of RNAi to identify genes regulating muscle protein degradation. (A) Chronic feeding of RNAi (Figure 1A) can be used to confirm that the RNAi feeding vector is working to produce the expected mutant phenotype, Dumpy (Dpy), and identify genes that when knocked down display less β-galactosidase reporter protein activity (blue) in muscle. (B) Feeding RNAi to fully developed adult animals (Figure 1B) can be used to determine if a gene product is normally required to prevent protein degradation in muscle. In this instance, UNC-82 prevents β-galactosidase degradation in all body-wall muscles. (C) Mutants can be used to confirm results from RNAi screens. These animals lack blue stain only in the head. All data are taken from the “muscle mutant” screen [30], images are all to the same scale with the exception of the mutant in (C); both scale bars are 100 µm.

We designed our primary screen to treat animals chronically with RNAi, scoring for reduced levels of the β-galactosidase reporter protein in two successive generations (Figure 1A). Genes whose knockdown decreased β-galactosidase staining (for example, *dpy-10* in Figure 2A) might encode either (a) products with a role in promoting expression of the reporter transgene, whether or not this role was transgene-specific [29], promoter-specific, or even muscle-specific or (b) products with a role in preventing abnormal protein degradation, again regardless of biochemical and/or cellular specificity. To distinguish between these alternatives, we then rescreened the genes identified in the multi-generation screen, for genes whose knockdown in adult animals caused short-term loss of the β-galactosidase staining in muscle (for example, *unc-82* in Figure 2B), an effect that can only reflect increased degradation of pre-existing reporter protein. 

At the time we started our work little was known about the efficacy of acute RNAi treatment of adult worms. In principle, a short-term RNAi treatment should produce a phenotype only if the targeted gene product is itself unstable, or more generally if continued synthesis of the target gene product is required to maintain a “normal” phenotype. For example, the latter situation obtains for the synthesis of dense body (attachment complex) proteins in muscle [25]. Acute RNAi knockdowns also bypass potential developmental effects (for example embryonic lethality [25]), permitting assessment of the roles of target gene products in the physiology of “normal” adults.

To test for effects of acute knockdown, we treated young adult worms over a period of 72 hours with RNAi against genes identified in the chronic screens (Figure 1B). For this secondary screen, we took replicates of 20 worms at 24, 48 and 72 hours after RNAi onset to test for reproducibility of the defects as well as for progressive decrease of β-galactosidase staining. This strategy allowed assessment of the efficacy of acute RNAi knockdown: Roughly 65% of the genes identified in our chronic screens also showed phenotypes when re-screened for acute, adult-onset RNAi effects. Without further experiments it is difficult to distinguish whether any gene for which RNAi treatment resulted in decreased β-galactosidase activity in the chronic screens but not in the acute screens is:

(a) A false positive in the chronic screen.
(b) A regulator of muscle protein synthesis but not degradation.
(c) A false negative (methodological error) in the acute screen.
(d) A regulator of protein degradation that does not show an acute RNAi response because adequate expression of the target gene had occurred prior to the RNAi treatment.

However, it is now clear that one could simply screen adults transferred to RNAi feeder bacteria, to identify potential negative regulators of muscle protein degradation, subject to the caveat that regulators of class (d) above might be missed. Acute treatment of adult worms with RNAi may be beneficial for automation of RNAi screens [31,32,33], as screens could be performed in a set time frame with less need for transferring worms to new food. Our data also suggest that defects in mitochondrial and sarcomere structure can also be determined in acute screens.

## 4. Methods Employed to Ensure Effectiveness of RNAi during Screening

As a quality control measure in our screens, we used bacterial feeding vectors from previously published studies and we compared the behavioural and developmental phenotypes we observed to those reported for the same bacterial RNAi feeding vector in WormBase [12]. The comparison of phenotypes observed by us and by others revealed that the irreproducibility of the RNAi feeding method we used was lower compared to other screens using the standard protocol [11,34]. In our screen of roughly 150 “muscle mutant” genes [30], our observations matched past observations for 57% of the clones tested, which suggests a high level of reproducibility when using the standard protocol. For this screen we also compared our results to the known mutant phenotype and found that almost all the RNAi phenotypes we observed matched the known phenotypes for mutations in the same gene. Thus, it is likely that most irreproducibility in RNAi feeding screens is procedural rather than due to inability of the RNAi to produce the known mutant phenotype (see next section). While we successfully used known mutant phenotypes as an indication of RNAi efficacy, it is well to remember that it will not be true in all instances that effective RNAi knockdown reproduces a mutant phenotype. RNAi-treated animals contain reduced amounts of normal gene product, whereas mutant animals may (depending on the particular allele) contain a normal amount of abnormal gene product. This may be an especially important consideration when the gene product participates in supramolecular assemblies, including those involved in signal transduction. Furthermore, mutations typically affect all cells that express the mutated gene (these in turn may affect other cells), whereas the efficacy of RNAi knockdown varies from tissue to tissue or possibly even cell to cell. For example, RNAi in *C. elegans* has relatively little effect on gene expression in neurons [35], which may contribute to discrepancies between mutant and RNAi phenotypes. However, in our screens, we viewed this is an advantage for identifying non-neuronal genes that affect muscle physiology. Others may prefer to use a mutant with increased sensitivity to RNAi [7,9,14,35] as an additional measure to ensure effectiveness of RNAi during screening. When making such a choice, it is well to remember that so far we know little about whether or how such mutants have altered expression of other genes in consequence of their altered RNA metabolism.

For our screens of the kinases and phosphatases we also employed a positive control. We used RNAi knockdown of *unc-112* (which encodes the adhesion site protein Kindlin) [36] as it produces both an obvious behavioural phenotype (paralysis) and subcellular defects (altered sarcomere and mitochondrial structure and activation of protein degradation) [25]. This control was run with every batch of genes examined (typically groups of 5–25 genes were assessed together). A lack of paralysis or lack of evident defects on the subcellular level indicated that the RNAi feeding in a particular experimental batch was not fully effective, thus prompting us to rerun the whole batch of genes examined. While the goal is to ensure that the RNAi results from each experimental batch are based on a similar RNAi effectiveness, there still remain the aforementioned problems with inability to precisely dose the knockdown and worm to worm differences (*i.e*., the penetrance of RNAi [7]). For example, in our “muscle mutant” screen [30] RNAi against *unc-112* resulted in tears in the normal array of sarcomeres whereas our follow up work on *unc-112* and other genes [25] demonstrated a reproducible collapse of sarcomeres, a much more severe phenotype than observed during the multi-gene screen. The apparent penetrance of the more severe subcellular phenotype was improved by choosing only those animals that showed a strong behavioural phenotype (as a proxy for strength of RNAi effect), whereas in the multi-gene screen no bias was used to select worms for analysis of subcellular defect. (Note that behavioural phenotypes can be independent of specific subcellular phenotypes.) Clearly, use of some method to select individuals that have received a more effective knockdown can increase confidence in the efficacy of knockdown. While we did not do this in our screens, others might consider using feeding vectors expressing dsRNA from two gene sequences in a single plasmid [16] to select animals more affected by RNAi against the ‘indicator’ gene (N.B. this expedient carries with it the hazard that the ‘indicator’ phenotype may influence the phenotype of interest). 

## 5. Methods Employed to Reduce False Negatives During RNAi Screening

In an effort to lower the frequency of false negatives and potentially reduce variability typically observed in the standard protocol [11,34], in the primary screen we applied RNAi over multiple generations of worms (this included a second exposure to fresh dsRNA in the second generation) and scored phenotypes multiple times (Figure 1A). In the second and third generations, animals (multiple replicates of 20 worms) were observed on the first day of adulthood and also 24 hours later. This enabled us to determine if phenotypes (developmental, behavioural, and/or subcellular defects) were reproducible within one and/or across two generations. These repeated observations not only make it less likely that a phenotype is simply missed, but also improve the acuity of individual observers through practice. For example, while a dumpy phenotype can look quite obvious once it has been observed (Figure 2A) it may take several observations of a population to note a dumpy phenotype that is present in a population at a lower penetrance (e.g. <50% of animals). Similarly, some phenotypes can be subtle and are only recognized after repeated observation. For example, *unc-82* treated animals look relatively wild-type (Figure 2B) but move more slowly whereas *unc-23* treated animals (Figure 2C) often appear to push their heads to one side when moving forward but look relatively normal when moving in reverse. While we did not use automated methods these can be employed to monitor various phenotypes and do so in an easily quantified fashion [31,32,33,37,38,39,40,41,42,43] thereby also potentially reducing false negatives during RNAi screening. 

Our protocol also enabled us to detect potential progressive effects in response to RNAi knockdown. That is, an increase in severity of a phenotype from day one to day two of adulthood in both generations of the primary screen or the secondary screen (Figure 1B) indicated that the phenotype observed was convincing. For example, if protein degradation is triggered, then total reporter protein should decrease with time. Those instances in which we observed defects in both generations in the primary screen provided the most convincing gene identifications. However, we also scored an RNAi effect if both replicates in only one generation showed defects or if defects were observed in only one replicate per generation but in both generations. This approach allowed us to take into account the following possibilities:

(a) The RNAi might not have been efficient in one of the generations.
(b) Not all the animals on the plate were affected by RNAi.
(c) The most affected animals of a generation may have been selected and scored on day 1, thus introducing selection bias into the day 2 observations.

In our screen of “muscle mutant” genes [30], only 3% of RNAi treatments failed to reproduce a known phenotype. We found a similarly low false negative rate in our (unpublished) screens of the full genomic sets of protein kinases (3%) and phosphatases (4%). This is much lower than the 30%–50% false negative rates in RNAi screens of others [3,10,11,35]. We believe that we achieved these much lower rates of false negatives because of the combined effect of the *unc-112* RNAi control in each batch of feedings and prior knowledge of behavioural and/or developmental phenotypes to decide if batches of genes needed to be re-examined (as discussed in the previous section), as well as our increased number of replicate observations. While our protocol appears quite effective, more replicates require greater time and cost, thus potentially limiting the number of genes that can be examined with finite resources.

## 6. Using Multiple Phenotypes to Increase Mechanistic Understanding

A common approach in systems biology is to use correlative analysis with large datasets to form hypotheses about underlying mechanisms and/or gene products that act together to influence a phenotype of interest [3]. Thus, in addition to assaying behavioural and developmental phenotypes and protein degradation we also assayed mitochondrial and sarcomere structure in response to RNAi. At the most simplistic level, a gene identified by RNAi as a potential regulator of protein degradation alone is likely acting via a mechanism distinct from that of a gene identified as a potential regulator of both protein degradation and mitochondrial and/or sarcomere structure. Using this approach, we found in all three screens that more genes appeared to regulate protein degradation alone than to regulate multiple subcellular processes. However, there are some clear differences between the sets of genes screened. For example, in the “muscle mutant” screen there was an over-representation of genes for which knockdown affected protein degradation and sarcomere and mitochondrial structure [30], whereas in the phosphatase screen there was almost an equal likelihood that a gene regulated protein degradation alone as protein degradation and mitochondrial structure. This clustering based on the coincidence of multiple subcellular phenotypes allows the investigator to choose sets of genes that may have related functions. For example, in following up the over-representation of genes that appeared to regulate protein degradation and sarcomere and mitochondrial structure, we identified a novel network of genes that regulate protein degradation in *C. elegans* muscle [25]. The decision to follow up these genes was also taken based upon bioinformatics and use of mutants and drug (see below).

The observation of defects in multiple subcellular processes may also serve as another internal control of RNAi efficacy. For example, if a gene is identified as a regulator of both mitochondrial structure and protein degradation in a chronic screen but only of one process in an acute screen, it may be that there is a false positive in the chronic screen or a false negative in the acute RNAi screen. These RNAi experiments could be repeated to test for reproducibility. We have used this coincidence analysis in our studies. 

## 7. Using Bioinformatics to Gain Further Mechanistic Insight and to Identify Potential False Negatives

Another common approach used in systems biology is to use data from others in addition to using one’s own data to perform correlative analysis [3]. Such data mining and use is commonly termed using bioinformatics. We employed five commonly used sets of data: gene ontology, known physical interactions between gene products, predicted physical interactions between gene products, known functional interactions, and predicted functional interactions. 

Gene ontologies are terms assigned to a gene [44]. These terms, referred to as GO terms, describe the gene products’ known role in a biological process, known molecular function, or known (sub-)cellular location. GO terms are intended to help to standardize gene descriptions across species and can be useful in quickly assessing what is known about the gene product. We used the *C. elegans* database (www.wormbase.org, [12]) to access gene ontologies, as well as other knowledge of specific genes of interest. For the sets of genes we identified as regulating muscle protein degradation alone or in combination with other subcellular processes, we used the Database for Annotation, Visualization, and Integrated Discovery (DAVID) [45] to look for enriched gene ontologies. Using this approach we found that for the “muscle mutant” screen [30] the GO term *locomotion* was enriched. This suggests that a future RNAi screen of the other roughly 1,000 genes that have been assigned the GO term *locomotion* might turn up additional regulators of muscle protein degradation. Based upon extrapolation from the initial screen [30] this is predicted to be roughly 250 additional genes. However, gene ontology analysis depends on the current knowledge about the identified genes, so it was not particularly surprising that for the screen of protein kinase genes, roughly 50% of the genes we identified as regulators of protein degradation returned without a GO term (other than protein phosphorylation). Thus, using a gene ontology analysis one can either decide to focus on genes that have been assigned a specific GO term or largely unstudied genes for further mechanistic insight. In theory, mechanistic insight can be gained faster by studying the genes with well studied GO terms but insights from this set are likely to be less novel than those from the unstudied genes.

Network analysis involves construction of protein-protein interaction or gene regulatory networks using bioinformatics [3]. Networks are used to visualize the relationships between genes and/or gene products. The gene or gene product is commonly described as a node and is often depicted as a circle in images generated using programs such as Cytoscape [46,47]. Links between genes can be established using many types of information. For example, in our screens we used known physical interactions (yeast two hybrid data), predicted physical interactions (data for the orthologues of the genes in yeast, flies, rodents, and/or man), known functional interactions (genetic), and predicted functional interactions (data for the orthologues of the genes in yeast, flies, rodents, and/or man). Each link between two genes, or nodes, is termed an edge and is often depicted as a line connecting two circles. To construct our networks we used data from WormBase [12], GeneMANIA [48,49] and Phosphopoint [50]. These network representations provide testable hypotheses of mechanisms regulating muscle protein degradation. For example, in the “muscle mutants” screen [30] several genes were found to form a network of physically interacting genes that included a ligand, a receptor, several intracellular binding partners, and a potential regulatory gene (*dim-1* [51]). We therefore investigated this predicted model [25] using traditional epistasis testing with mutants and drugs as described below. 

In addition to formulating testable hypotheses, bioinformatics can be exploited to increase confidence in the RNAi results obtained. Genes for which RNAi knockdown induces cytosolic protein degradation should, largely, act with other gene products in order to regulate muscle protein degradation. Thus, GO and network analyses provide candidate genes that interact with identified genes. If an identified gene is bioinformatically linked to another gene for which RNAi did not induce protein degradation (or other phenotype(s) of interest), one could retest the negative result to potentially determine if it was a false negative. For example, in our “muscle mutant” screen [30] *unc-97* and *unc-112* were not identified as regulating mitochondrial network structure, whereas a set of physically associated proteins were so identified. Subsequent retesting [25] of *unc-97* and *unc-112* revealed that they were false negatives in the initial screen. As a general principle, two genes whose products interact in a regulatory network should give the same knockdown phenotype, if and only if their actions as regulators have the same sense; there should be no such coincidence if one gene product is a positive regulator and the other a negative regulator.

## 8. Using Mutants and Drugs for Confirmation and Mechanistic Insight

**Figure 3:**
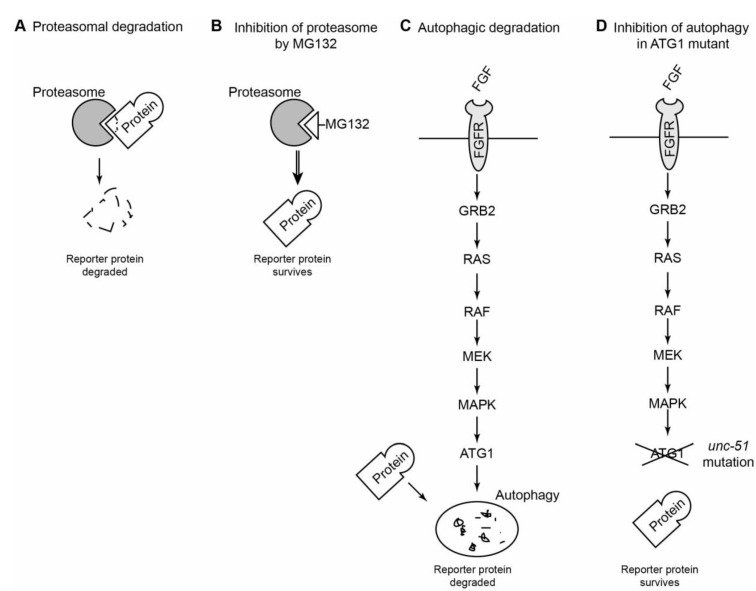
Examples of use of drugs and mutants to identify what protease is required for muscle protein degradation in response to RNAi against a gene of interest. (A) Schematic of a proteasome degrading a protein. (B) Schematic of a proteasome inhibitor blocking the ability of the proteasome to degrade a protein. (C) Schematic of Fibroblast Growth Factor (FGF) signalling inducing autophagy in *C. elegans* muscle [26,27,28]. (D) Schematic of how a mutation in *unc-51* blocks autophagy in response to FGF signalling [26,28] or signalling that impinges upon FGF signalling [27].

The *C. elegans* model for understanding genetic regulation of muscle protein degradation was constructed using traditional genetic and pharmacologic techniques [21,24,26,27,28]. Thus, the mutations and drugs identified as inhibitors of muscle protein degradation are now available as tools for testing if novel genes identified as potential regulators of degradation act through known or novel signalling pathways (Figure 3). At the simplest level, if a mutation or drug treatment prevents a given RNAi from inducing a phenotype, the gene targeted by the RNAi acts ‘upstream’ of the mutational or drug blockade. For example, if RNAi against a gene induces protein degradation in a wild-type animal (Figure 3C) but not in an *unc-51* mutant (Figure 3D) then the targeted gene likely acts upstream of *unc-51* (for example it could act at the level of RAF in Figure 3D). If the mutation or drug is without effect, the gene targeted by RNAi lies ‘downstream’ or acts in a different pathway altogether. For example, if RNAi against a gene induces protein degradation in a wild-type animal (Figure 3C) and in an *unc-51* mutant (not shown) then the targeted gene likely acts downstream of *unc-51* to influence autophagy or RNAi against the gene acts to cause non-autophagic protein degradation. It is crucial to keep in mind that this epistasis test can be interpreted in such a simple fashion only if the step blocked by mutation or drug is *necessary* for the RNAi-induced pathology. For example, if RNAi against a gene induces protein degradation and the proteasome inhibitor MG132 blocks degradation in response to RNAi (Figure 3B) then the proteasome is required for the observed degradation. However, the failure of a mutation or drug to prevent an RNAi-induced pathology means that the step blocked by mutation or drug is not necessary for that pathology; it may or may not be involved. This point is especially crucial for processes such as protein degradation, where multiple mechanisms (e.g., proteasome, lysosomes, calpains, caspases) may act in parallel under some circumstances.

To implement this method, once we identified a gene as a potential negative regulator of muscle protein degradation, we performed a tertiary screen where we acutely treated mutant or drug treated adult animals with RNAi against a candidate gene (Figure 1C). Note that this was only done for those genes where sensitivity to acute (adult-onset) RNAi showed that there must be an effect on degradation (Figure 1B, Figure 2B) rather than on reporter protein synthesis and/or degradation (Figure 2A). In the tertiary screens we included wild-type adults as a control, to confirm that RNAi against the candidate gene did induce degradation and followed the same time course as in the secondary screen. For the tertiary screen, unlike the secondary screen, we also included a replicate and in the case of discrepant results we ran a second replicate. Thus the epistasis testing, during which acute RNAi knockdown was rerun on control animals, also served as another internal control to report on the false positive rate of the acute RNAi screen. Interestingly, whereas the false positive rate for developmental and behavioural phenotype was 1% or less in each of our screens, the potential false positive rate for identification of genes regulating muscle protein degradation was 8%, 15%, and 4% respectively for our screens of “muscle mutants” [30], protein kinases, and phosphatases, respectively. It is not clear if the false-positive frequency increased because of variability in the efficacy of the RNAi treatment (we estimate variability at 6%–10% for behavioural and developmental phenotypes), or because our methodology decreased the false negative rate, thus biasing in favour of a false positive. It may also be that subcellular phenotypes show more natural variation. For example, we [25] and others [52] see mitochondrial defects in 20%–30% of non-RNAi treated animals. 

Data from the mutant and drug treated animals also provided mechanistic insight. For example, in the screen of “muscle mutants” [30], RNAi against the majority of identified genes induced degradation that was not blocked in mutant or drug treated animals. These results suggest that we identified either (a) a large number of regulators acting in previously unknown networks or (b) a large number of regulators acting downstream of the mutational or drug blocks we used or (c) genes whose knockdown triggers multiple proteolytic mechanisms. In contrast, in the screens of protein kinases and phosphatases, RNAi against the majority of identified genes failed to induce degradation in *mpk-1* (MAPK) or in *unc-51* (autophagy-related protein 1) mutant animals. These results suggest that the majority of identified protein kinases and phosphatases are regulators of autophagy via a MAPK signalling cascade. Thus, the use of mutants and drugs for epistasis testing allows the investigator to classify genes of interest as either potential novel regulators of protein degradation or genes acting in a novel way within a known signalling network. By combining this additional information with the previously discussed network models, one can directly begin to study either set of genes.

We have used this approach to test and confirm one predicted new proteolytic mechanism in muscle [25]. Several genes, including *unc-52*, *unc-97*, and *unc-112* were identified as potential novel regulators of muscle protein degradation, as inducing multiple identical phenotypes, and as physically interacting [30]. We therefore knocked down the remaining proteins that were known to interact with these physically and/or functionally, and confirmed that the integrin receptor and its associated proteins are negative regulators of muscle protein degradation. We further showed that mutation in a functionally interacting gene, *dim-1*, previously identified as a suppressor of *unc-112* [51], suppressed degradation not only in *unc-112* RNAi treated animals but also in response to RNAi against each of the integrin interacting genes. This experiment established that a common mechanism was underlying the degradation observed in response to each of the 14 RNAi treatments studied. We next confirmed the *unc-112* RNAi results with a temperature-sensitive *unc-112* mutant. The induction of mitochondrial defects in this mutant allowed us to definitively resolve the discrepancy between the initial RNAi screen [30] where *unc-112* RNAi did not disrupt mitochondrial network structure, and the subsequent RNAi follow up [25] in which it did so. Similarly, mutants can also be used to confirm that observed phenotypes in RNAi screening are limited to specific muscles (for example, *unc-23* in Figure 2C). The *unc-112* mutant also provided a new tool with which new drug and RNAi treatments could be used to identify the relevant protease and drugs. RNAi targeting any one of several calpain genes was found to block degradation in *unc-112* mutants. In addition to validating a hypothesis generated from RNAi screening and bioinformatics, these additional experiments provided two new tools (*dim-1* mutants and calpain inhibitors), which can be added to tertiary screening as discussed above. Thus, a full cycle of discovery feeding back into improved RNAi screening methodology can be achieved via this approach. 

## 9. Conclusions

The false negative rate in *C. elegans* RNAi screens by feeding experiments can be reduced by using previously developed RNAi feeding vectors, coupled with a positive control for RNAi efficacy, replicates, and multiple rounds of RNAi treatment. Assaying multiple phenotypes in parallel not only increases reliability, but also may provide mechanistic insight. Further insight into mechanism can be provided by the use of bioinformatics and by additional rounds of screening in mutant background, or in the presence of drugs, to establish epistasis relationships.
